# Prevalence and associated factors for dipstick microscopic hematuria in men

**DOI:** 10.1186/s12894-019-0505-1

**Published:** 2019-08-06

**Authors:** Karim Courtemanche, Peter Chan, Wassim Kassouf

**Affiliations:** Division of Urology, McGill University Health Center, 1001 Blvd Decarie, room E02.4366, Montreal, Quebec H4A 3J1 Canada

**Keywords:** Microscopic hematuria, Prevalence, Risk factors

## Abstract

**Background:**

Microscopic hematuria is a common incidental finding on routine urinalysis. Although there are no clear recommendations to perform routine urinalysis, some studies have shown that up to 50% of general practitioners continue to perform annual routine urinalysis regardless of age or risk factors. The aim of this study was to identify associated factors and prevalence of dipstick microscopic hematuria in the general male population presenting to an annual public men’s health fair.

**Method:**

We conducted a retrospective analysis of prospectively collected data at an annual Men’s Health fair from 2008 to 2013. Patient reported health questionnaires, basic physical exam including digital rectal exam, basic bloodwork and dipstick urinalysis data was examined.

**Results:**

A total of 979 patients were reviewed. Of these, 850 provided a urine sample and were included in the final analysis. Seventy-three (8.6%) patients had positive hematuria on urinalysis. Average age in both groups was 55 years. Presence of microscopic hematuria was correlated with presence of diabetes and proteinuria with odds-ratio of 2.8 (1.3–5.8) and 2.9 (1.7–5.0) respectively on multivariate analysis. There was no significant correlation identified with age, hypertension, coronary artery disease, body-mass index, smoking, prostate specific antigen (PSA) or International Prostate Symptom Score (IPSS). Limitation of this study is the lack of follow-up and knowledge of subsequent investigations of patients.

**Conclusion:**

Microscopic hematuria is a prevalent condition in the male population presenting to a health fair. The only factors associated with microscopic hematuria were diabetes and proteinuria. No association was found between hematuria and smoking, age, or lower urinary tract symptoms.

## Background

Many etiologies exist for microscopic hematuria such as infection, urolithiasis, benign prostate hyperplasia, malignancy, nephropathies or physiologic. About 3.6% of patients presenting with microscopic hematuria will present a urinary tract malignancy [1] but positive predictive value of microhematuria for malignancy is low and thus population screening has not been recommended [1–3]. The definition of microscopic hematuria is three or greater red-blood cells (RBC) per high power field on a properly collected microscopic urinalysis as per the American Urological Association guidelines [1] whereas the Canadian Urological Association guidelines require confirmation with a second microscopic urinalysis [4]. However, in clinical practice, microscopy is often not ordered or not performed by the laboratory. Several studies have confirmed that dipstick urinalysis has good specificity (65–95%) and sensitivity (91–100%) in detecting microhematuria; [5] rates of false positives and negatives are low and can be due to hemoglobinuria, myoglobinuria, ascorbic acid or reducing agents such as povidone.

Routine screening dipstick urinalysis is currently not recommended by any organization, including the Canadian Task Force on Preventive Health Care, as there is insufficient data to support this type of screening [6]. However, up to 50% of general practitioners perform a routine urinalysis on all their patients as part of annual health screening visits [7]. Microscopic hematuria (MH) is a common finding during routine urinalysis and leads to frequent referrals to urologists and nephrologists [8]. According to current guidelines, MH often prompts a workup of the lower and upper tracts with cystoscopy and abdominal imaging representing a significant cost to health care systems [4].

Our primary objective was to identify and describe factors associated with microscopic hematuria. We also report on the prevalence of MH in the male population presenting to a men’s health fair.

## Methods

Men who presented to an annual McGill University public men’s health fair held in public venues in Montreal, Canada from 2008 to 2013 were included. Informed consents were obtained from all participants to permit anonymous analyses of collected data for educational and academic research purposes. All men who presented to this annual health fair were eligible for inclusion into the study. In addition to physical examination, patients were asked to complete a health questionnaire including socio-demographic data, medical history, medications, lifestyle (smoking, alcohol use and drugs), Metabolic Equivalent of Task scale (METs), Sexual Health Inventory for Men (SHIM), Androgen Deficiency in Aging Male (ADAM), Berlin questionnaire for obstructive sleep apnea (BQ), International Prostate Symptom Score (IPSS) and Overactive Bladder 8 (OAB8). Depending on their age, patients were offered prostate specific antigen (PSA), cholesterol panel and testosterone level testing. They also underwent a digital rectal exam and a dipstick urinalysis.

Patients who stated a previous history of hematuria or did not provide a urine sample were excluded from the final study population. Baseline characteristics were compared using t-tests for continuous variables and chi-square or Fisher exact for categorical variables depending on the number of patients in that particular analysis. Multivariate logistic regression was used to examine various factors and their association with microscopic hematuria and establish the odds-ratio of microhematuria on dipstick urinalysis. Where data was missing, analysis was done only with patients with complete records available. Missing data was primarily due to patients not answering specific questions on the questionnaire. Significant *p* values were set as two-tailed *p* < 0.05. Data was analyzed using SPSS version 17.

## Results

Data was reviewed on 979 patients who presented to the men’s health fair from 2008 to 2013; 850 provided a urine sample and had no history of previous hematuria and formed the basis of this report. Mean and median age of men was 54.7 and 55 respectively (range 20 to 87). Baseline demographics of the population are presented in Table 1 Mean body-mass index (BMI) was 27.6. Of 850 patients, 67 (7.9%) had diabetes mellitus (DM), 46 (5.4%) had coronary artery disease (CAD), 156 (18.4%) had hypertension (HTN) and 18 (2.1%) had a history of malignancy. From a urological aspect, 42 (4.9%) had a history of previous urological surgery, 42 (4.9%) had a history of urinary or urological infections and 41 (4.8%) had a history of stones.

**Table 1:** Demographics and comorbidities

Age in year (range)	55 (20–87)
Education Level	Grade 7 or lower: 3.1% (26)
	Some high school: 8.9% (76)
	High school graduate or CEGEP: 24.8% (211)
	Some University: 13.1% (111)
	University Graduate: 37.2% (316)
	Post-graduate studies: 11.4% (97)
Smoking history	Non-smoker: 67.9% (573)
	Smoker: 12.3% (104)
	Ex-smoker: 19.8% (167)
BMI (kg/m^2^)	27.6 ± 5.0
Dipstick hematuria	Overall: 73 (8.6%)
	+: 41 (4.8%)
	++: 17 (2%)
	+++: 11 (1.3%)
	++++: 4 (0.5%)
Proteinuria	128 (15.1%)
Diabetes	67 (7.9%)
Hypertension	156 (18.4%)
Coronary artery disease	46 (5.4%)
PSA (ng/mL)	1.54 ± 1.74

Seventy-three (8.6%) patients demonstrated MH. There was no significant difference in rates of coronary artery disease, previous history of urolithiasis, malignancy, urological surgery, urological infections or hypertension between patients with and without MH. Smoking rates (present, past, ever) were similar in both groups (Table 2). Distribution of IPSS score categories was similar between both groups (6.1% vs 6.3% severe scores, *p* = 0.825). There was no difference between SHIM score categories (*p* = 0.479). On multivariable analysis, the only factors associated with MH were diabetes (OR 2.8, *p* = 0.007) and proteinuria (OR 2.9, *p* = 0.0001) (Table 3).

**Table 2:** Comparison of associated factors

Characteristic	No hematuria (*n* = 777)	Microhematuria (*n* = 73)	N	p
Age	54.7 (+/−12.9)	55.4 (+/−10.6)	850	0.669
Diabetes mellitus	7.1% (55)	16.4%(12)	850	0.005
Hypertension	18.3% (142)	19.2% (14)	850	0.849
Coronary artery disease	5.4% (42)	5.5% (4)	850	1.000
Malignancy	2.2% (17)	1.4% (1)	850	1.000
Urological surgery	4.8% (37)	6.8% (5)	850	0.431
History of urolithiasis	4.8% (37)	5.5% (4)	850	0.773
Urological infections	5.1% (40)	2.7% (2)	850	0.571
Smoking	12.4% (96)	11.3% (8)	844	0.778
Proteinuria	13.4% (104)	32.9% (24)	850	0.0001
IPSS category (mild, mod, severe)	Mild: 58% (407)	Mild: 61.9% (39)	765	0.825
	Moderate: 35.9% (252)	Moderate: 31.7% (20)		
	Severe: 6.1% (43)	Severe: 6.3% (4)		
OAB-q v8	39.6% (280)	34.3% (23)	774	0.434
SHIM	No ED: 48% (306)	No ED: 53.2% (33)	797	0.479
	Mild: 28.7% (183)	Mild: 27.4% (17)		
	Mild-Mod: 3.3% (85)	Mild-Mod: 8.1% (5)		
	Moderate: 6.1% (39)	Moderate: 9.7% (6)		
	Severe: 3.9% (25)	Severe: 1.6% (1)		
ADAM questionnaire positive	64.4% (482)	52.9% (37)	819	0.069
Metabolic syndrome	28.5% (142)	39.4% (13)	532	0.234
PSA > 4 ng/ml	7.3% (47)	7.5% (5)	715	0.950
BMI	< 25: 28.8% (220)	< 25: 26.0% (19)	837	0.688
	25–30: 45.5% (348)	25–30: 43.8% (32)		
	> 30: 25.7% (196)	> 30: 30.1% (22)

**Table 3:** Multivariate logistic regression analysis of factors associated with microhematuria

Variable	OR	p
Diabetes	2.758 (1.312–5.799)	0.007
Hypertension	0.775 (0.383–1.568)	0.478
Coronary artery disease	0.965 (0.318–2.932)	0.951
Smoking	0.934 (0.427–2.045)	0.864
Proteinuria	2.882 (1.669–4.976)	0.0001
BMI ≥ 30	1.015 (0.966–1.068)	0.551
Age	0.998 (0.977–1.020)	0.857

### Discussion

This study demonstrated that microscopic hematuria is prevalent in the male population and diabetes is the primary factor associated with increased risk of detecting MH on dipstick urinalysis.

Multiple studies evaluated the prevalence of microscopic hematuria with varying rates in a general screening population. Our observed prevalence of microscopic hematuria was 8.6% which is similar to other studies in men ranging from 2.5% [9] to 13% [10]. Previous studies have examined the risk factors of urinary tract malignancy diagnosed following workup of hematuria. Risk factors of malignancy identified in some of these studies were age, smoking, the presence of gross hematuria and positive cytology [11–13]. Kang et al. found in a screening study that male sex and diabetes increased the risk of finding underlying pathology on workup of MH [12]. None of the studies identified in our review of the literature analyzed factors associated with likelihood of detecting MH. In our study, a past medical history of diabetes increased the odds of hematuria by 2.8 times. Okada et al. showed that microscopic hematuria had little clinical significance in diabetic nephropathy and that creatinine doubling time or increase of serum creatinine was no different between patients with persistent MH or without MH [14]. Hematuria was also significantly associated with the presence of concomitant proteinuria with an odds ratio of 2.9, likely associated with the presence of diabetes. The increase risk of finding MH in diabetic patients especially in the context of proteinuria could be used to better counsel patients when these abnormal results are found.

How would the identification of factors associated with MH help counselling the patient? The yield of finding malignancy in patients with MH is very low. AUA meta-analysis found that the incidence of malignancy upon workup of asymptomatic MH was 3.3% when based on one positive urine sample [1]. According to current guidelines, MH prompts a workup of the lower and upper tracts with cystoscopy and abdominal imaging representing a significant cost to health care systems. [4] Further prospective studies are needed to evaluate whether the identification of factors associated with increased risk of MH may help stratifying patients who can obviate investigations with invasive procedures.

Some limitations exist in our study. All past medical history was self-reported by patients and may thus induce a bias. The population presenting to this health fair may not be representative of the general population as patients with specific concerns or who do not have regular medical follow-up may have been overrepresented. However, the prevalence of smoking and comorbidities such as hypertension, diabetes, and coronary artery disease was similar to rates in the general Canadian population [15, 16]. Furthermore, this was a single institution experience and this may limit generalizability of our findings. Another limitation is that the dipstick microhematuria was not confirmed with microscopy in this study. Several studies have confirmed that dipstick urinalysis has good specificity (65–95%) and sensitivity (91–100%) in detecting microhematuria [5, 17] but should still be confirmed with microscopy before ordering costly and invasive testing. Lastly and importantly, follow-up data was not available for our patient population to identify if these patients ultimately underwent full workup and their findings.

## Conclusion

The prevalence of microhematuria in the general male population presenting to a public health fair was 8.6%, in keeping with other reported studies of prevalence of MH. Significant factors associated with MH were a history of diabetes and concomitant proteinuria.

## Data Availability

The datasets used and/or analysed during the current study available from the corresponding author on reasonable request.

## Abbreviations

ADAM: Androgen Deficiency in Aging Male
BMI: Body-mass index
BQ: Berlin questionnaire
CAD: Coronary artery disease
DM: Diabetes mellitus
HTN: hypertension
IPSS: International prostate symptom score
METs: Metabolic equivalent of task scale
MH: Microscopic hematuria
OAB8: Overactive bladder 8
PSA: Prostate specific antigen
RBC: Red-blood cells
SHIM: Sexual Health Inventory for Men
